# Ostrowski and Čebyšev type inequalities for interval-valued functions and applications

**DOI:** 10.1371/journal.pone.0291349

**Published:** 2023-09-25

**Authors:** Jing Guo, Xianjun Zhu, Wenfeng Li, Hui Li

**Affiliations:** 1 School of Software Engineering, Jinling Institute of Technology, Nanjing, P. R. China; 2 School of Engineering Management, Nanjing University, Nanjing, P. R. China; 3 Reproductive Medicine Center, Jinling Hospital, Nanjing, P. R. China; 4 Research Center for New Technology in Intelligent Equipment Nanjing University, Nanjing, P. R. China; 5 College of Computer Science and Technology, Nanjing University of Aeronautics and Astronautics, Nanjing, P. R. China; National Kaohsiung University of Science and Technology / Industrial University of Ho Chi Minh, TAIWAN

## Abstract

As an essential part of classical analysis, Ostrowski and Čebyšev type inequalities have recently attracted considerable attention. Due to its universality, the non-additive integral inequality takes several forms, including Sugeno integrals, Choquet integrals, and pseudo-integrals. Set-valued analysis, a well-known generalization of classical analysis, is frequently employed in studying mathematical economics, control theory, etc. Inspired by pioneering work on interval-valued inequalities, this paper establishes specific Ostrowski and Čebyšev type inequalities for interval-valued functions. Moreover, the error estimation to quadrature rules is presented as some applications for illustrating our results. In addition, illustrative examples are offered to demonstrate the applicability of the mathematical methods presented.

## Introduction

Interval-valued functions hold immense mathematical and practical significance, occupying a central role across diverse academic disciplines. Their distinct properties and characteristics are integral to the study of interval optimization, interval differential equations, and random set analysis. In particular, interval-valued functions exhibiting integrability and differentiability play a crucial role in the abovementioned areas. Furthermore, these functions assume a prominent position within the fuzzy theory, as they enable the representation of fuzzy-valued functions through a collection of interval-valued functions, utilizing the notion of levels within a fuzzy interval. The main topic of this paper is Ostrowski and Čebyšev type inequalities. In 1882, Čebyšev [1] obtained an inequality:

$$\begin{array}{c} |\frac{1}{b-a}\int_{a}^{b}f(t)g(t)dt-\frac{1}{{\left(b-a\right)}^{2}}\int_{a}^{b}f(t)dt\int_{a}^{b}g(t)dt|\leq \frac{{\left(b-a\right)}^{2}}{12}\|f^{\prime }{\|}_{\infty }{\left\|g^{\prime }\right\|}_{\infty }, \end{array}$$ (1)

where f,g:[a,b]→R are absolutely continuous functions, and *f*′, *g*′ ∈ *L*_∞_([*a*, *b*]).

In 1938, Ostrowski [2] proved the following inequality:

$$\begin{array}{c} |f\left(x\right)-\frac{1}{b-a}\int_{a}^{b}f\left(t\right)dt|\leq \frac{\|f^{\prime }{\|}_{\infty }}{b-a}[\frac{{\left(b-a\right)}^{2}}{4}+{\left(x-\frac{a+b}{2}\right)}^{2}], \end{array}$$ (2)

where f:[a,b]→R be a differentiable mapping in (*a*, *b*), and *f*′ ∈ *L*_∞_([*a*, *b*]).

Integral inequalities of the Ostrowski, Chebyshev, and Gruss type are well-known and appear in many areas of mathematics(for history and generalizations, see the renowned monograph [3], along with the papers [4–7]). Čebyšev and Ostrowski type inequalities, which have a very close relationship (see details in [8]), play an important role in many areas of mathematical applications and have received extensive attention from researchers. For example, Grüss [9] presented an inequality as follows:

$$\begin{array}{c} |\frac{1}{b-a}\int_{a}^{b}f(t)g(t)dt-\frac{1}{{\left(b-a\right)}^{2}}\int_{a}^{b}f(t)dt\int_{a}^{b}g(t)dt|\leq \frac{1}{4}\left(\Phi -\phi \right)\left(\Gamma -\gamma \right), \end{array}$$ (3)

where f,g:[a,b]→R be two integrable functions with *ϕ* ≤ *f*(*x*) ≤ Φ, *γ* ≤ *g*(*x*) ≤ Γ for all *x* ∈ [*a*, *b*] and ϕ,Φ,γ,Γ∈R. Ujević [10] obtained the following Ostrowski type inequality:

$$\begin{array}{c} |f\left(x\right)-\frac{1}{b-a}\int_{a}^{b}f\left(t\right)dt-\frac{f(b)-f(a)}{b-a}(x-\frac{a+b}{2})|\leq \frac{\sqrt{\left(b-a\right)\|f^{\prime }{\|}_{2}^{2}-{\left(f\left(b\right)-f\left(a\right)\right)}^{2}}}{2\sqrt{3}}, \end{array}$$ (4)

where f:[a,b]→R be a differentiable mapping with *f*′ ∈ *L*_2_[*a*, *b*]. The constant $\frac{1}{2\sqrt{3}}$ is the best possible. For details of other Čebyšev and Ostrowski type inequalities, we refer the interested reader to [11–20].

Constructing a variety of integral inequalities is a contemporary concern. In recent years, a great amount of meaningful work has been completed using a variety of integrals, such as the Sugeno integral [21, 22], the pseudo integral [23], and the Choquet integral [24], etc. Interval-valued functions [25], as a concept of generalization of functions and an important mathematical topic, have become an increasingly vital instrument for solving practical issues, particularly in mathematical economics [26]. Recent research has extended some traditional integral inequalities to the domain of interval- valued functions. Costa et al. [27] presented new interval versions of Minkowski and Beckenbach’s integral inequalities. Hermite Hadamard, Jensen, and Ostrowski-type inequalities were proven in this generalization [28]. Also, Hermite-Hadamard and Hermite-Hadamard-type inequalities were addressed using interval-valued Riemann-Liouville fractional integrals [29]. Zhao et al. [30–32] studied Chebyshev type inequalities, Opial-type integral inequalities, and Jensen and Hermite-Hadamard type inequalities for interval-valued functions using the gH -differentiable or h-convex concept. Budaka et al. [33] derived novel fractional inequalities of the Ostrowski type for interval-valued functions, utilizing the definitions of gH-derivatives. Khan et al. [34] introduced log-h-convex fuzzy-interval-valued functions as a distinct class of convex fuzzy-interval-valued functions, employing a fuzzy order relation. This class served to establish Jensen and Hermite-Hadamard inequalities.

Incorporating the Ostrowski-type inequality within the realm of fuzzy-valued functions necessitated the utilization of the Hukuhara derivative, as demonstrated by Anastassiou [35]. Fuzzy-valued functions, also known as functions with an interval value, were central to Anastassiou’s [35] research. Fascinatingly, the fuzzy Ostrowski-type inequalities derived by Anastassiou [35] also extended their validity to interval-valued functions. For a comprehensive understanding of the constraints imposed by the concept of the H-derivative on interval-valued functions, it is worth examining the works of Bede and Gal [36] and Chalco-Cano et al. [37]. Notably, recent contributions by Chalco-Cano et al. [38] have successfully established an Ostrowski type inequality catering specifically to generalized Hukuhara differentiable interval-valued functions. The utmost significance of generalized Hukuhara differentiability as the most comprehensive concept for characterizing the differentiability of interval-valued functions has been underscored in prominent studies by Bede and Gal [36], Chalco-Cano et al. [39].

To the best of my knowledge, there is a gap in the literature regarding exploring Čebyšev and Ostrowski type inequalities utilizing generalized Hukuhara differentiation. Motivated by the existing literature, our primary objective is to introduce a novel approach that addresses the challenges associated with interval-valued functions. Specifically, we aim to present a series of interval-based variations of Čebyšev and Ostrowski type inequalities, utilizing the concept of generalized Hukuhara differentiability (as referenced in [39, 40–42]). This proposed approach offers several significant benefits:

Enhanced Precision: By developing interval versions of Čebyšev and Ostrowski-type inequalities, our research enables a more precise analysis of interval-valued functions. This heightened precision can be precious in scientific and engineering fields where accuracy is paramount.

Improved Handling of Uncertainty: Interval-valued functions inherently capture uncertainty in data or model parameters. Using generalized Hukuhara differentiability, our methodology provides a robust framework for effectively managing this uncertainty. It mainly benefits decision-making processes, risk assessment, and optimization under uncertain conditions.

Broadened Applicability: The introduction of interval versions of these well-known inequalities expands the applicability of the existing theory to interval-valued functions. This extension not only enriches the field of interval analysis but also allows for new avenues of research and exploration of mathematical properties specific to interval-valued operations.

Estimating error in quadrature rules for gH-differentiable interval-valued functions is a fundamental application of these newly derived inequalities. The first application of Ostrowski’s inequality to quadrature formulas was given in [43].(see also [44]). This paper also presented new quadrature procedures and error estimates as a theoretical application of this new fuzzy Čebyšev and Ostrowski type inequalities. When our goal is to obtain definite integrals of the form $\int_{a}^{b}f\left(t\right)dt=\left[\int_{a}^{b}\underline{f(t)}dt,\int_{a}^{b}\bar{f(t)}dt\right]$ in (8), classical quadrature rules, such as the trapezoidal and Simpson’s rules, can be employed to approximate $\int_{a}^{b}f\left(t\right)dt=\int_{a}^{b}\underline{f(t)}dt$ and $\int_{a}^{b}f\left(t\right)dt=\int_{a}^{b}\bar{f(t)}dt$. The research conducted by Chalco-Cano et al. [37] has highlighted that gH-differentiability in *f* does not imply the gH-differentiability of $\underline{f}$ and $\bar{f}$. Consequently, the conventional error estimation methods that rely on differentiability cannot be directly utilized for these quadrature rules. In contrast, when working with interval-valued functions represented by *f*, leveraging established interval arithmetic techniques and software allows for approximating the definite integral $\int_{a}^{b}f\left(t\right)dt$. For a comprehensive understanding of this approach, refer to the studies conducted by Moore [45] and Moore et al. [46]. Given the widespread prevalence of interval-valued functions, acquiring the capability to estimate their errors and proficiently employ quadrature techniques becomes indispensable.

The study is organized as follows: Section 2 presents some relevant preliminaries. Section 3 presents a new Čebyšev type inequality and two Ostrowski type inequalities. Section 4 discusses the new estimation of quadrature rules which contain the mid-point quadrature rule as a special case based on the results in Section 3.

## Preliminaries

Let R be the real line, and let $R_{I}$ denote the family of all closed interval of R, that is,

$$R_{I}=\left\{\left[a,b\right]\;|\;a,b\in R\;\text{and}\;a\leq b\right\}.$$

If *a* = *b*, then the interval [*a*, *b*] is reduced to a real number a∈R, i.e., a real number a∈R could be regarded as a degenerated interval [*a*, *a*].

Let $A=[\underline{a},\bar{a}]$ and $B=[\underline{b},\bar{b}]$, the interval arithmetic are defined as follows:

- *Addition*:
  $$A+B=[\underline{a}+\underline{b},\bar{a}+\bar{b}];$$
- *Multiplication*:
  $$A\times B=AB=[\text{min}\left\{\underline{a}\;\underline{b},\underline{a}\bar{b},\bar{a}\underline{b},\bar{a}\bar{b}\right\},\text{max}\left\{\underline{a}\;\underline{b},\underline{a}\bar{b},\bar{a}\underline{b},\bar{a}\bar{b}\right\}];$$
- *Generalized Hukuhara difference (gH-difference)* [39, 40–42]:
  $$A{⊖}_{gH}B=\left[\text{min}\left\{\underline{a}-\underline{b},\bar{a}-\bar{b}\right\},\text{max}\left\{\underline{a}-\underline{b},\bar{a}-\bar{b}\right\}\right].$$

Now, we impose the Hausdorff metric or distance on $R_{I}$, that is,

$$H\left(A,B\right)=\text{max}\{|\underline{a}-\underline{b}\left|,\right|\bar{a}-\bar{b}\left|\right\},\;\forall A,B\in R_{I}.$$

**Lemma 1** [47] H *is a complete metric in* $R_{I}$ *and has the following properties*

*(i)* $H\left(A,B\right)=H\left(A{⊖}_{gH}B,0\right),$

*(ii)* H(λA,λB)=|λ|H(A,B),λ∈R,

*(iii)* H(A+C,B+D)≤H(A,B)+H(C,D).

Note that for any interval-valued function $f:\left[a,b\right]\to R_{I}$, there exists two real functions $\underline{f},\bar{f}$ with $\underline{f}\leq \bar{f}$ on [*a*, *b*] such that

$$\begin{array}{c} f\left(t\right)=[\underline{f}\left(t\right),\bar{f}\left(t\right)],\;\forall t\in \left[a,b\right] \end{array}.$$ (5)

The functions $\underline{f}$ and $\bar{f}$ are called the lower and the upper functions of *f*, respectively.

We say an interval-valued function *f* is continuous at *t*_0_ ∈ [*a*, *b*] if both $\underline{f}$ and $\bar{f}$ are continuous functions at *t*_0_ ∈ [*a*, *b*]. Denote by $C(\left[a,b\right],R_{I})$ the space of all continuous interval-valued functions. Then, $C(\left[a,b\right],R_{I})$ is a quasilinear space (see [47]) endowed with a quasi-norm ‖ ⋅ ‖_∞_ given by

$$\begin{array}{c} {\left\|f\right\|}_{\infty }=\underset{t\in [a,b]}{\text{sup}}\;H\left(f\left(t\right),0\right). \end{array}$$ (6)

**Definition 2** [48] *Let* $f:\left[a,b\right]\to R_{I}$ *be an interval-valued function*. *f is said to be Aumann integrable if the set S*(*f*) *of all integrable selectors of f, i.e*.,

$$S\left(f\right)=\{\hat{f}:\left[a,b\right]\to R|\hat{f}\left(x\right)\in f\left(x\right)\;a.e.\;is\;integrable\}$$

*is nonempty. The Aumann integral of f over* [*a*, *b*] *is defined as*

$$\begin{array}{c} \int_{a}^{b}f\left(t\right)dt=\{\int_{a}^{b}\hat{f}\left(t\right)dt\;|\;\hat{f}\in S\left(f\right)\}. \end{array}$$ (7)

**Lemma 3** [48] *Let* $f,g:\left[a,b\right]\to R_{I}$ *be two measurable and integrability bounded interval-valued functions. Then for any* [*c*, *d*] ⊂ [*a*, *b*],

*(i)* $\int_{c}^{d}\left(\alpha f\left(t\right)+\beta g\left(t\right)\right)dt=\alpha \int_{c}^{d}f\left(t\right)dt+\beta \int_{c}^{d}g\left(t\right)dt,\;\forall \alpha ,\beta \in R;$

*(ii)* $\int_{c}^{d}f\left(t\right)dt=\int_{c}^{\tau }f\left(t\right)dt+\int_{\tau }^{d}f\left(t\right)dt,\;\forall \tau \in \left[c,d\right];$

*(iii)* $H(\int_{c}^{d}f\left(t\right)dt,\int_{c}^{d}g\left(t\right)dt)\leq \int_{c}^{d}H\left(f\left(t\right),g\left(t\right)\right)dt$ .

**Definition 4** [26] *Let* $f:\left[a,b\right]\to R_{I}$ *be an interval-valued function such that* $f\left(t\right)=\left[\underline{f(t)},\bar{f(t)}\right]$. *Then*, $\underline{f(t)}$,$\bar{f(t)}$ *are integrable functions defined as*

$$\begin{array}{c} \int_{a}^{b}f\left(t\right)dt=\left[\int_{a}^{b}\underline{f(t)}dt,\int_{a}^{b}\bar{f(t)}dt\right]. \end{array}$$ (8)

**Definition 5** [48] *Let* $f:\left[a,b\right]\to R_{I}$ *be an interval-valued function, we say that f is generalized Hukuhara differentiable (gH-differentiable) at t*_0_ ∈ [*a*, *b*] *if*

$$\begin{array}{c} f{}^{\prime }\left(t_{0}\right)≔\underset{h\to 0}{\text{lim}}\;\frac{f\left(t_{0}+h\right){⊖}_{gH}\;f\left(t_{0}\right)}{h} \end{array}$$ (9)

*exists, and f*′(*t*_0_) *is called the gH-derivative of f at t*_0_.

**Lemma 6** [42]. *Let* $f:\left[a,b\right]\to R_{I}$ *be a gH-differentiable interval-valued function. If its gH-derivative f*′ *is continuous, then*

$$\begin{array}{c} H\left(f\left(d\right),f\left(c\right)\right)\leq \left(d-c\right)\underset{t\in [c,d]}{\text{sup}}\;H\left(f{}^{\prime }\left(t\right),0\right), \end{array}$$ (10)

*for any* [*c*, *d*] ⊂ [*a*, *b*].

## Inequalities for interval-valued functions

This section presents Ostrowski and Čebyšev type inequalities for continuously gH-differentiable interval-value functions. First, we offer an Ostrowski type inequality.

**Theorem 7** *Let* $f:\left[a,b\right]\to R_{I}$ *be an interval-value function. If its gH-derivative f*′ *is continuous, then for any x* ∈ [*a*, *b*],

$$\begin{array}{c} H(f(x){⊖}_{gH}\frac{1}{b-a}\int_{a}^{b}f(t)dt,\frac{f(b){⊖}_{gH}\;f(a)}{b-a}(x-\frac{a+b}{2}))\\ \leq \frac{\text{max}\{{\left(x-a\right)}^{2},{\left(x-b\right)}^{2}\}}{b-a}{\left\|f{}^{\prime }\right\|}_{\infty }. \end{array}$$

*Proof* According to Lemmas 1 and 3, we have

$$\begin{array}{c} H(f(x){⊖}_{gH}\frac{1}{b-a}\int_{a}^{b}f(t)dt,\frac{f(b){⊖}_{gH}\;f(a)}{b-a}(x-\frac{a+b}{2}))\\ =H(f(x){⊖}_{gH}\frac{1}{b-a}\int_{a}^{b}f(t)dt+0,0+\frac{f(b){⊖}_{gH}\;f(a)}{b-a}(x-\frac{a+b}{2}))\\ \leq H(f(x){⊖}_{gH}\frac{1}{b-a}\int_{a}^{b}f(t)dt,0)+H(0,\frac{f(b){⊖}_{gH}\;f(a)}{b-a}(x-\frac{a+b}{2}))\\ =H(f(x),\frac{1}{b-a}\int_{a}^{b}f(t)dt)+H(\frac{f(b){⊖}_{gH}\;f(a)}{b-a}(x-\frac{a+b}{2}),0). \end{array}$$ (11)

Furthermore, due to [49] [Theorem 4], one has

$$\begin{array}{c} H(f(x),\frac{1}{b-a}\int_{a}^{b}f(t)dt)\leq \frac{1}{b-a}[\frac{{\left(b-a\right)}^{2}}{4}+{\left(x-\frac{a+b}{2}\right)}^{2}]{\left\|f{}^{\prime }\right\|}_{\infty }. \end{array}$$ (12)

On the other hand,

$$\begin{array}{c} \begin{array}{cc} H(\frac{f(b){⊖}_{gH}\;f(a)}{b-a}(x-\frac{a+b}{2}),0) & =\frac{\left|x-\frac{a+b}{2}\right|}{b-a}H(f(b){⊖}_{gH}\;f(a),0)\\ & \leq |x-\frac{a+b}{2}|{\left\|f^{{}^{\prime }}\right\|}_{\infty }. \end{array} \end{array}$$ (13)

Thus, it follows from (11)–(13) that

$$\begin{array}{c} H(f(x){⊖}_{gH}\frac{1}{b-a}\int_{a}^{b}f(t)dt,\frac{f(b){⊖}_{gH}\;f(a)}{b-a}(x-\frac{a+b}{2}))\\ \leq \frac{\text{max}\{{\left(x-a\right)}^{2},{\left(x-b\right)}^{2}\}}{b-a}{\left\|f{}^{\prime }\right\|}_{\infty }. \end{array}$$

This ends the proof.

**Example 8** *Considering f*(*t*) = [*t*, *t*^2^], *t* ∈ [1, e]. *Then* ‖*f*′‖_∞_ = 2e, *and*

$$A\left(x\right)≔f(x){⊖}_{gH}\frac{1}{e-1}\int_{1}^{e}f(t)dt=\left[x,x^{2}\right]{⊖}_{gH}[\frac{e+1}{2},\frac{e^{2}+e+1}{3}],$$

$$B\left(x\right)≔\frac{f(e){⊖}_{gH}\;f(1)}{e-1}(x-\frac{e+1}{2})=(x-\frac{e+1}{2})\left[1,e+1\right].$$

*Then*,

$$\begin{array}{c} H\left(A\left(x\right),B\left(x\right)\right)=\{\begin{array}{cc} -x^{2}+x+\frac{2e^{2}-e-1}{6}, & \;if\;x\in (\frac{e+1}{2},c],\\ ex-\frac{e^{2}+e}{2}, & \;if\;x\in (c,d],\\ |x^{2}-\left(e+1\right)x+\frac{e^{2}+4e+1}{6}|, & \;if\;x\in \left[1,e\right]\backslash (\frac{e+1}{2},d], \end{array} \end{array}$$

*where*

$$c=\frac{3-3e+\sqrt{39e^{2}-6e+3}}{6},\;d=\frac{3+\sqrt{12e^{2}-6e+3}}{6}.$$

*On the other hand*,

$$\begin{array}{c} E\left(x\right)≔\frac{\text{max}\{{\left(x-1\right)}^{2},{\left(x-e\right)}^{2}\}}{e-1}{\left\|f{}^{\prime }\right\|}_{\infty }=\{\begin{array}{cc} \frac{2e}{e-1}{\left(x-e\right)}^{2},\; & \;if\;x\in [1,\frac{e+1}{2}],\\ \frac{2e}{e-1}{\left(x-1\right)}^{2},\; & \;if\;x\in (\frac{e+1}{2},e]. \end{array} \end{array}$$

**Fig 1:**
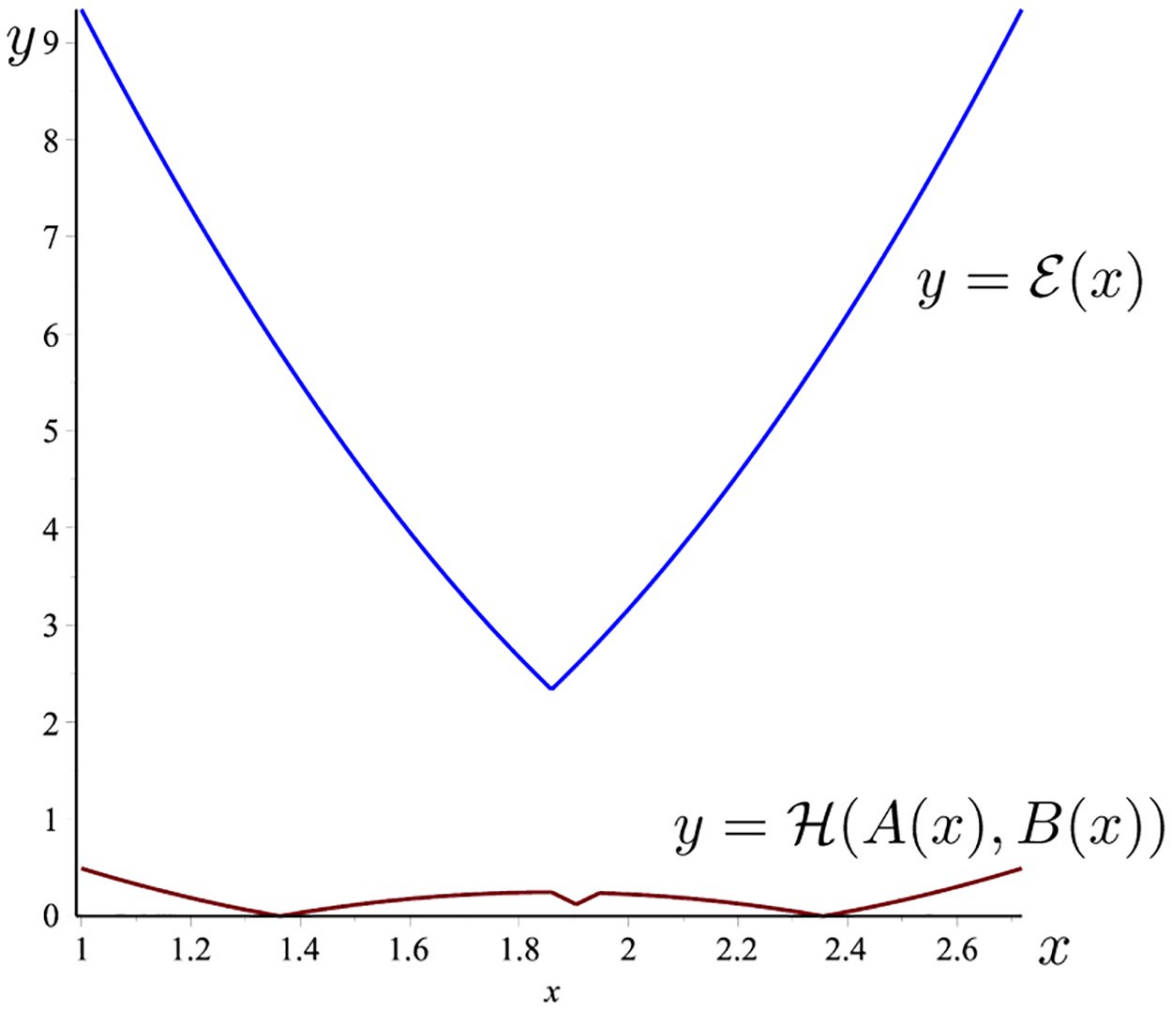
H(A(x),B(x)) (the red line) and E(x) (the blue line) given in Example 8.

*The graph of the two functions* H(A(x),B(x)) *and* E(x) *is given in* Fig 1. *It is easy to see that*

H(A(x),B(x))≤E(x),∀x∈[1,e].

*So that the theorem 7 holds*.

In Theorem 7, if $x=\frac{a+b}{2}$, then we present another one generalization of inequality.

**Corollary 9** *Let* $f:\left[a,b\right]\to R_{I}$ *be an interval-value function. If its gH-derivative f*′ *is continuous, then*

$$\begin{array}{c} H(f(\frac{a+b}{2}),\frac{1}{b-a}\int_{a}^{b}f(t)dt)\leq \frac{b-a}{4}{\left\|f{}^{\prime }\right\|}_{\infty }. \end{array}$$ (14)

**Example 10** *Let f*(*t*) = [*t*^2^, *t*^3^], *t* ∈ [1, e]. *It is easy to see that f*′(*t*) = [2*t*, 3*t*] *on* [1, e] *with* ‖*f*′‖_∞_ = 3e^2^. *Moreover*,

$$A≔f\left(\frac{1+e}{2}\right)=\left[{\left(\frac{e+1}{2}\right)}^{2},{\left(\frac{e+1}{2}\right)}^{3}\right],$$

$$B≔\frac{1}{e-1}\int_{1}^{e}f(t)dt=\left[\frac{e^{2}+e+1}{3},\frac{\left(e^{2}+1\right)\left(e+1\right)}{4}\right].$$

*Hence*,

$$H\left(A,B\right)=\text{max}\left(\frac{{\left(e^{2}-1\right)}^{2}}{12},\frac{e^{3}-e^{2}-e+1}{8}\right),$$

$$\varepsilon =\frac{3}{4}\left(e^{3}-e\right).$$

*This ends the proof*.

According to Theorem 7, we have

**Lemma 11** *Let* $f:\left[a,b\right]\to R_{I}$ *be an interval-value function*, g:[a,b]→R *a bounded function. If f has a continuous gH-derivative f*′, *then for any x* ∈ [*a*, *b*],

$$H(f(x)g(x),\frac{1}{b-a}\int_{a}^{b}f(t)dt\cdot g(x))$$

$$\leq \frac{1}{b-a}[\frac{{\left(b-a\right)}^{2}}{4}+{\left(x-\frac{a+b}{2}\right)}^{2}]{\left\|g\right\|}_{\infty }{\left\|f{}^{\prime }\right\|}_{\infty }.$$

**Example 12** *Considering f*(*t*) = [*t*, 2*t*], *g*(*t*) = 2^*t*^, *t* ∈ [0, 1]. *Then* ‖*f*′‖_∞_ = 2, ,‖*g*‖_∞_ = 2, *and*

$$A\left(x\right)≔f\left(x\right)g\left(x\right)=\left[x,2x\right]2^{x}=\left[x2^{x},2x2^{x}\right],$$

$$B\left(x\right)≔\int_{0}^{1}\left[t,2t\right]dt2^{x}=\left[\frac{1}{2}2^{x},2^{x}\right].$$

*Then*,

$$H\left(A\left(x\right),B\left(x\right)\right)=|2^{x}\left(2x-1\right)|.$$

*On the other hand*,

$$E\left(x\right)=4\left(\frac{1}{4}+{\left(x-\frac{1}{2}\right)}^{2}\right)=4x^{2}-4x+2.$$

**Fig 2:**
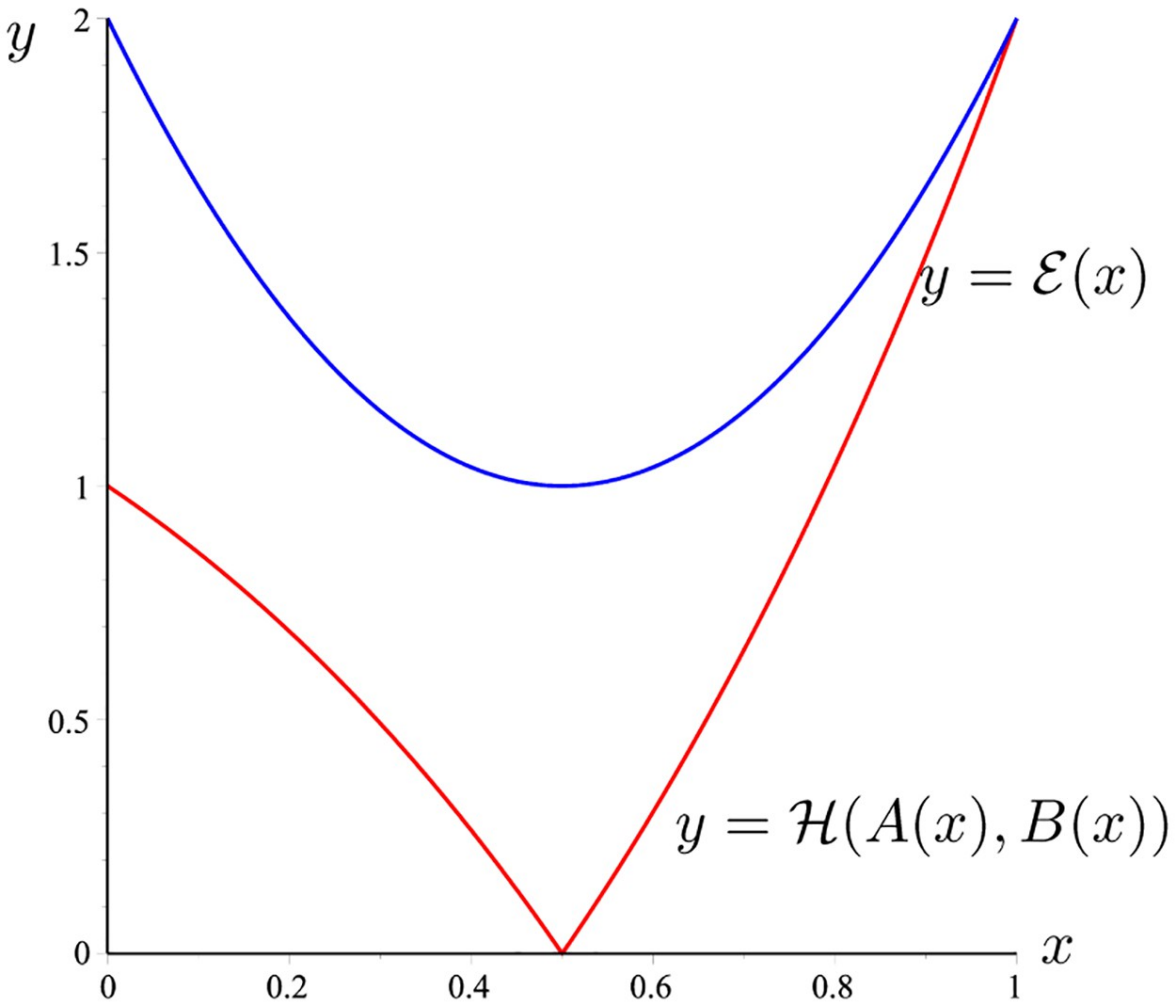
H(A(x),B(x)) (the red line) and E(x) (the blue line) given in Example 12.

*The graph of the two functions* H(A(x),B(x)) *and* E(x) *is given in* Fig 2. *It is easy to see that*

H(A(x),B(x))≤E(x),∀x∈[0,1].

*So the lemma 11 is proved*.

**Theorem 13** *Let* $f:\left[a,b\right]\to R_{I}$ *be an interval-value function with a continuous gH-derivative f*′. *If* g:[a,b]→R *is an absolutely continuous function, and g*′ ∈ *L*_∞_([*a*, *b*]), *then for any x* ∈ [*a*, *b*],

$$\begin{array}{c} \begin{array}{cc} & H(f(x)g(x),\frac{1}{{\left(b-a\right)}^{2}}\int_{a}^{b}f(t)dt\int_{a}^{b}g(t)dt)\\ \leq & \frac{1}{b-a}[\frac{{\left(b-a\right)}^{2}}{4}+{\left(x-\frac{a+b}{2}\right)}^{2}]{\left(\|g\right\|}_{\infty }\|f{}^{\prime }{\|}_{\infty }+\|g^{\prime }{\|}_{\infty }{\left\|f\right\|}_{\infty }). \end{array} \end{array}$$

*Proof* By Lemma 1, one has

$$\begin{array}{c} \;H(f(x)g(x),\frac{1}{{\left(b-a\right)}^{2}}\int_{a}^{b}f(t)dt\int_{a}^{b}g(t)dt)\\ \leq H(f(x)g(x),\frac{1}{b-a}\int_{a}^{b}f(t)dt\cdot g(x))\\ \;+H(\frac{1}{b-a}\int_{a}^{b}f(t)dt\cdot g(x),\frac{1}{{\left(b-a\right)}^{2}}\int_{a}^{b}f(t)dt\int_{a}^{b}g(t)dt). \end{array}$$ (15)

Due to Lemma 11,

$$\begin{array}{c} H(f(x)g(x),\frac{1}{b-a}\int_{a}^{b}f(t)dt\cdot g(x))\leq \frac{1}{b-a}[\frac{{\left(b-a\right)}^{2}}{4}+{\left(x-\frac{a+b}{2}\right)}^{2}]{\left\|g\right\|}_{\infty }{\left\|f{}^{\prime }\right\|}_{\infty }. \end{array}$$ (16)

On the other hand, from (2), it follows that

$$\begin{array}{c} \;H(\frac{1}{b-a}\int_{a}^{b}f(t)dt\cdot g(x),\frac{1}{{\left(b-a\right)}^{2}}\int_{a}^{b}f(t)dt\int_{a}^{b}g(t)dt)\\ \leq \left|g\left(x\right)-\frac{1}{b-a}\int_{a}^{b}g(t)dt|H(\frac{1}{b-a}\int_{a}^{b}f(t)dt,0\right)\\ \leq \frac{1}{b-a}[\frac{{\left(b-a\right)}^{2}}{4}+{\left(x-\frac{a+b}{2}\right)}^{2}]\|g^{\prime }{\|}_{\infty }{\left\|f\right\|}_{\infty }. \end{array}$$ (17)

Hence,

$$\begin{array}{c} \;H(f(x)g(x),\frac{1}{{\left(b-a\right)}^{2}}\int_{a}^{b}f(t)dt\int_{a}^{b}g(t)dt)\\ \leq \frac{1}{b-a}[\frac{{\left(b-a\right)}^{2}}{4}+{\left(x-\frac{a+b}{2}\right)}^{2}]{\left(\|g\right\|}_{\infty }\|f{}^{\prime }{\|}_{\infty }+\|g^{\prime }{\|}_{\infty }{\left\|f\right\|}_{\infty }). \end{array}$$ (18)

This ends the proof.

**Example 14** *Considering* *f*(*t*) = [*t*^2^, *t*^3^],$g(t)=\frac{1}{t}$, *t* ∈ [1, 2]. *Then* ‖*f*′‖_∞_ = 12, ,‖*g*‖_∞_ = 1, ‖*f*|_∞_ = 8, ,‖*g*′‖_∞_ = 1, *and*

$$A\left(x\right)≔f\left(x\right)g\left(x\right)=\left[x^{2},x^{3}\right]\frac{1}{x}=\left[x,x^{2}\right],$$

$$B\left(x\right)≔\int_{1}^{2}\left[t^{2},t^{3}\right]dt\int_{1}^{2}\frac{1}{t}dt=\left[\frac{7}{3}\text{ln}\;2,\frac{15}{4}\text{ln}\;2\right].$$

*Then*,

$$H\left(A\left(x\right),B\left(x\right)\right)=|x^{2}-\left(15/4\right)*ln\left(2\right)|.$$

*On the other hand*,

$$E\left(x\right)=20\left(\frac{1}{4}+{\left(x-\frac{3}{2}\right)}^{2}\right)=10\left(2x^{2}-6x+5\right).$$

**Fig 3:**
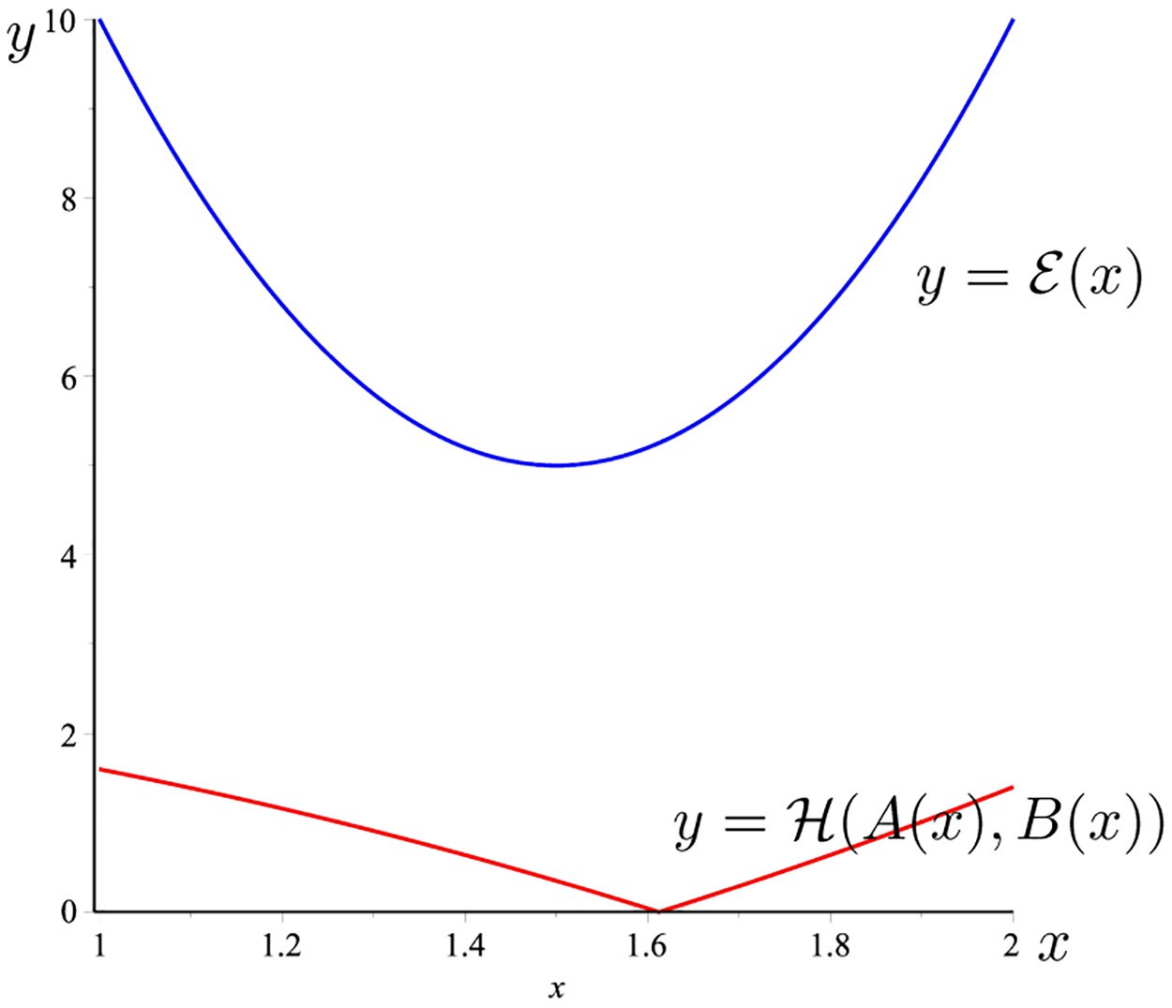
H(A(x),B(x)) (the red line) and E(x) (the blue line) given in Example 14.

*The graph of the two functions* H(A(x),B(x)) *and* E(x) *is given in* Fig 3. *It is easy to see that*

H(A(x),B(x))≤E(x),∀x∈[1,2].

Now we are coming to the Čebyšev type inequality for interval-valued functions.

**Theorem 15** *Let* $f:\left[a,b\right]\to R_{I}$ *be an interval-value function*, g:[a,b]→R *a bounded function. If f has a continuous gH-derivative f*′, *then*

$$H(\frac{1}{b-a}\int_{a}^{b}f(t)g(t)dt,\frac{1}{{\left(b-a\right)}^{2}}\int_{a}^{b}f(t)dt\int_{a}^{b}g(t)dt)\leq \frac{\left(b-a\right)}{3}{\left\|g\right\|}_{\infty }{\left\|f{}^{\prime }\right\|}_{\infty }.$$

*Proof* By Lemmas 1 and 3, we have

$$\begin{array}{c} \;H(\frac{1}{b-a}\int_{a}^{b}f(t)g(t)dt,\frac{1}{{\left(b-a\right)}^{2}}\int_{a}^{b}f(t)dt\int_{a}^{b}g(t)dt)\\ =H(\frac{1}{{\left(b-a\right)}^{2}}\int_{a}^{b}\int_{a}^{b}f(t)g(t)dtdx,\frac{1}{{\left(b-a\right)}^{2}}\int_{a}^{b}\int_{a}^{b}f(x)g(t)dtdx)\\ \leq \frac{1}{{\left(b-a\right)}^{2}}\int_{a}^{b}H(\int_{a}^{b}f(t)g(t)dt,\int_{a}^{b}f(x)g(t)dt)dx\\ \leq \frac{1}{{\left(b-a\right)}^{2}}\int_{a}^{b}\int_{a}^{b}H(f(t)g(t),f(x)g(t))dtdx\\ =\frac{1}{{\left(b-a\right)}^{2}}\int_{a}^{b}\int_{a}^{b}\left|g(t)\right|H(f(t),f(x))dtdx. \end{array}$$ (19)

Moreover, according to Lemma 6, one has

$$\begin{array}{cc} \int_{a}^{b}\left|g\left(t\right)\right|H(f(t),f(x))dt & \leq \int_{a}^{b}\left|g\left(t\right)||x-t\right|(\underset{r\in [a,b]}{\text{sup}}\;H(f^{\prime }\left(r\right),0))dt\\ & \leq \int_{a}^{b}{\left\|g\right\|}_{\infty }|x-t|\|f{}^{\prime }{\|}_{\infty }dt\\ & ={\left\|g\right\|}_{\infty }{\left\|f{}^{\prime }\right\|}_{\infty }(x^{2}-\left(a+b\right)x+\frac{a^{2}+b^{2}}{2}). \end{array}$$ (20)

Thus, it follows from (19) and (20) that

$$\begin{array}{c} \;H(\frac{1}{b-a}\int_{a}^{b}f(t)g(t)dt,\frac{1}{{\left(b-a\right)}^{2}}\int_{a}^{b}f(t)dt\int_{a}^{b}g(t)dt)\\ \leq \frac{1}{{\left(b-a\right)}^{2}}\int_{a}^{b}\int_{a}^{b}\left|g(t)\right|H(f(t),f(x))dtdx\\ \leq \frac{1}{{\left(b-a\right)}^{2}}\int_{a}^{b}{\left\|g\right\|}_{\infty }{\left\|f{}^{\prime }\right\|}_{\infty }(x^{2}-\left(a+b\right)x+\frac{a^{2}+b^{2}}{2})dx\\ =\frac{\left(b-a\right)}{3}{\left\|g\right\|}_{\infty }{\left\|f{}^{\prime }\right\|}_{\infty }. \end{array}$$ (21)

According to Eq (21), the proof is therefore complete.

**Remark 16** *In Theorem 15, if g* ≡ 0 *on* [*a*, *b*], *then the equality holds*.

**Example 17** *Let f*(*t*) = [*t*, 2*t*], *g*(*t*) = e^*t*^, *t* ∈ [0, 1]. *It is easy to see that* *f*′(*t*) = [1, 2] *on* [0, 1] *with* ‖*f*′‖_∞_ = 2 *and* ‖*g*‖_∞_ = e. *Moreover*,

$$A≔\int_{0}^{1}f(t)g(t)dt=\int_{0}^{1}(\left[t,2t\right]\cdot e^{t})dt=\left[1,2\right],$$

$$B≔\int_{0}^{1}f(t)dt\int_{0}^{1}g(t)dt=\int_{0}^{1}\left[t,2t\right]dt\cdot \int_{0}^{1}e^{t}dt=[\frac{e}{2},e].$$

*Hence*,

$$H\left(A,B\right)=e-2\leq \frac{1}{3}\|f^{\prime }{\|}_{\infty }{\left\|g\right\|}_{\infty }=\frac{2}{3}e.$$

Thanks to Theorem 15, we have another Ostrowski type inequality.

**Corollary 18** *Let* $f:\left[a,b\right]\to R_{I}$ *be an interval-value function. If its second order gH-derivative f*″ *is continuous, then for any x* ∈ [*a*, *b*],

$$H(f(x){⊖}_{gH}\frac{1}{b-a}\int_{a}^{b}f(t)dt,\frac{f(b){⊖}_{gH}\;f(a)}{b-a}(x-\frac{a+b}{2}))\leq \frac{b^{2}-a^{2}}{6}{\left\|f{}^{\prime \prime }\right\|}_{\infty }.$$

*Proof* Let

$$u\left(t\right)=f^{\prime }\left(t\right){⊖}_{gH}\frac{1}{b-a}\int_{a}^{b}f^{\prime }\left(s\right)ds,$$

$$g\left(x,t\right)=K\left(x,t\right)-\frac{1}{b-a}\int_{a}^{b}K\left(t,s\right)ds,$$

where

$$K\left(x,t\right)=\{\begin{array}{cc} t-a,\; & t\in [a,x],\\ t-b, & t\in (x,b], \end{array}$$

From Lemmas 1 and 3 and Theorem 15, it follows that

$$\begin{array}{c} \;H(\frac{1}{b-a}\int_{a}^{b}u\left(t\right)g\left(x,t\right)dt,\frac{1}{{\left(b-a\right)}^{2}}\int_{a}^{b}u\left(t\right)dt\int_{a}^{b}g\left(x,t\right)dt)\\ =H(\frac{1}{b-a}\int_{a}^{b}u\left(t\right)g\left(x,t\right)dt,0)\\ =H(\frac{1}{b-a}\int_{a}^{b}g\left(x,t\right)f^{\prime }\left(t\right)dt,\frac{1}{b-a}\int_{a}^{b}g\left(x,t\right)\frac{f(b){⊖}_{gH}\;f(a)}{b-a}dt)\\ =H(f(x){⊖}_{gH}\frac{1}{b-a}\int_{a}^{b}f(t)dt,\frac{f(b){⊖}_{gH}\;f(a)}{b-a}(x-\frac{a+b}{2}))\\ \leq \frac{\left(b-a\right)}{3}{\left\|g\right\|}_{\infty }{\left\|u{}^{\prime }\right\|}_{\infty }\\ =\frac{b^{2}-a^{2}}{6}\left\|f^{\prime \prime }\right\|. \end{array}$$ (22)

According to (22), this ends the proof.

**Example 19** *Considering f*(*t*) = [*t*, *t*^2^], *t* ∈ [1, e]. *Then* ‖*f*″‖_∞_ = 2, *and*

$$\hat{E}\left(x\right)=\frac{e^{2}-1^{2}}{6}\left\|f^{\prime \prime }\right\|=\frac{e^{2}-1}{3},\;\forall x\in \left[1,e\right].$$

**Fig 4:**
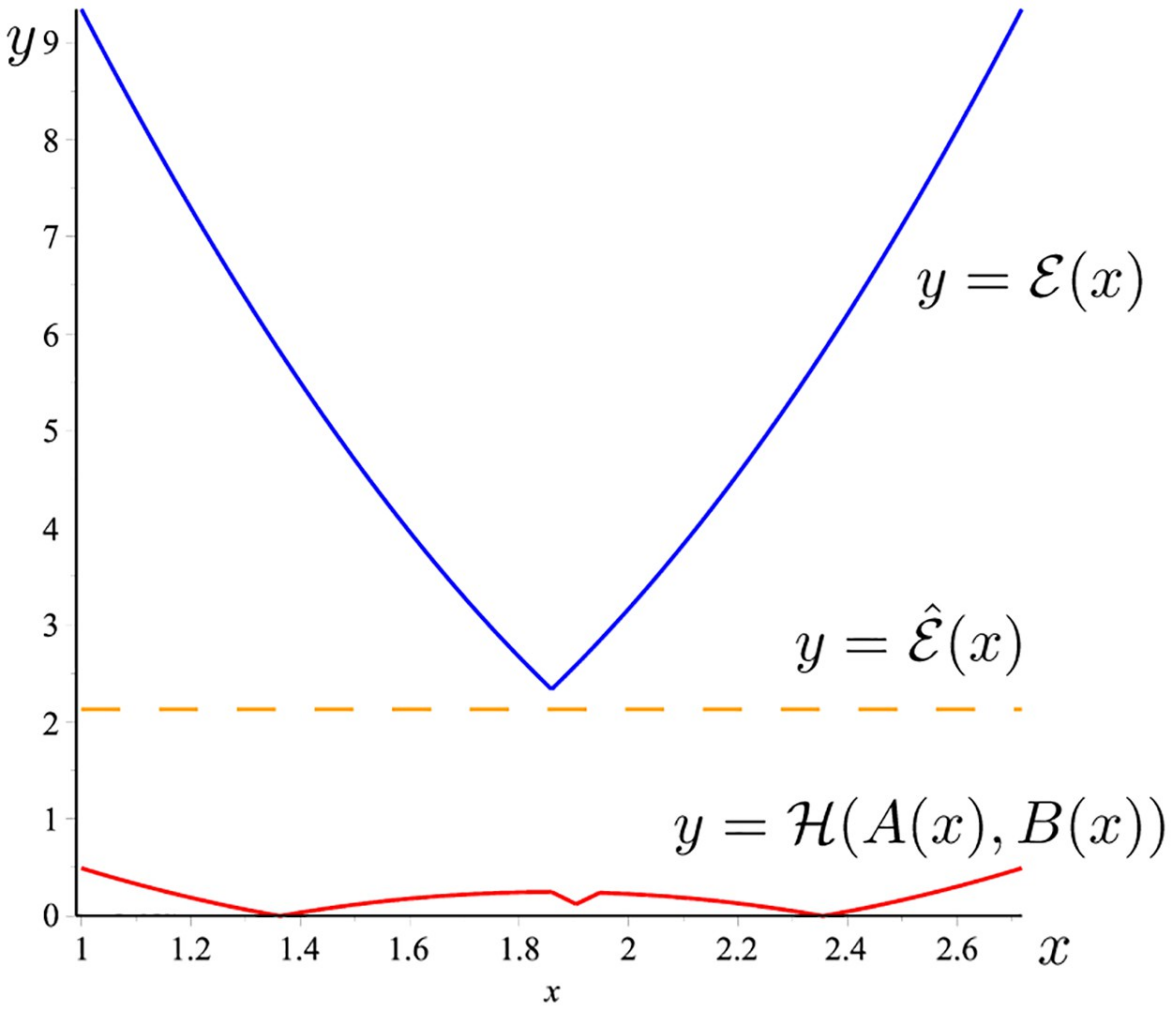
H(A(x),B(x)) (the red line), E(x) (the blue line) and $\hat{E}\left(x\right)$ (the dash line) given in Example 19.

*The graph of the three functions* H(A(x),B(x)), $\hat{E}\left(x\right)$ *and* E(x) *is given in* Fig 4. *It is easy to see that*

$$H\left(A\left(x\right),B\left(x\right)\right)\leq \hat{E}\left(x\right)\leq E\left(x\right),\;\forall x\in \left[1,e\right].$$

## Applications to the numerical quadrature rules

Let [a,b]⊂R, *I*_*h*_ : *a* = *t*_0_ < *t*_1_ < *t*_2_ < *t*_3_ < … < *t*_*n*_ = *b* is a partition of [*a*, *b*] with *h*_*i*_ = *t*_*i*_ − *t*_*i*−1_ (*i* = 1, 2, …, *n*), and *ξ* = (*ξ*_1_, *ξ*_2_, …, *ξ*_*n*_) is a intermediate point vector satisfying *ξ*_*i*_ ∈ [*t*_*i*−1_, *t*_*i*_] (*i* = 1, 2, …, *n*), then the finite collection of interval-point pairs (*I*_*h*_, *ξ*) is called a *K*-partition of [*a*, *b*]. Denote by $P(I_{h},\xi )$ the set of all *K*-partitions of [*a*, *b*].

**Proposition 20** *Let* $f:\left[a,b\right]\to R_{I}$ *be an interval-value function. If its second order gH-derivative f*″ *is continuous, then for any K-partition* $P\in P(I_{h},\xi )$,

$$H(A_{R}\left(f,I_{h},\eta \right),\int_{a}^{b}f(t)dt)\leq \frac{1}{4}\sum_{i=1}^{n}h_{i}^{2}{\left\|f{}^{\prime \prime }\right\|}_{\infty },$$

*where A*_*R*_ *denotes the quadrature rule of Riemann-type defined by*

$$A_{R}\left(f,I_{h},\eta \right)=\sum_{i=1}^{n}\;f(\eta_{i})h_{i},and\;\eta_{i}=\frac{t_{i}+t_{i-1}}{2}.$$

*Proof* From Lemmas 1 and Corollary 14, it follows that

$$\begin{array}{cc} H(A_{R}\left(f,I_{h},\eta \right),\int_{a}^{b}\;f(t)dt) & =H(\sum_{i=1}^{n}f\left(\eta_{i}\right)h_{i},\sum_{i=1}^{n}\int_{t_{i-1}}^{t_{i}}f(y)dy)\\ & =\sum_{i=1}^{n}H(f\left(\eta_{i}\right)h_{i},\int_{t_{i-1}}^{t_{i}}f(y)dy). \end{array}$$ (23)

According to [49, Theorem 4] and Eq (24), we have

$$\begin{array}{cc} \sum_{i=1}^{n}H(f\left(\eta_{i}\right)h_{i},\int_{t_{i-1}}^{t_{i}}f(y)dy) & \leq \|f{}^{\prime \prime }{\|}_{\infty }(\frac{{\left(\eta_{i}-t_{i-1}\right)}^{2}+{\left(t_{i}-\eta_{i}\right)}^{2}}{2})\\ & =\frac{1}{4}\sum_{i=1}^{n}{\left(h_{i}\right)}^{2}{\left\|f{}^{\prime \prime }\right\|}_{\infty }. \end{array}$$ (24)

The proof is therefore complete.

**Example 21** *Considering* *f*(*x*) = [*x*, *x*^2^ + 2*x*], *and* $t_{i}=\frac{i}{n}\left(i=1,2,...,n\right),h_{i}=\frac{1}{n}.$

*We can get* ‖*f*″‖_∞_ = 2, *and*

$$A_{R}=\sum_{i=1}^{n}\;f(\eta_{i})\frac{1}{n},where\;\eta_{i}=\frac{t_{i}+t_{i-1}}{2}.$$

*Thus, we have*

$$A_{R}=\sum_{i=1}^{n}\left[\frac{2i-1}{2n},{\left(\frac{2i-1}{2n}\right)}^{2}+2\left(\frac{2i-1}{2n}\right)\right]\cdot \frac{1}{n}=\left[\frac{1}{2},\frac{4}{3}-\frac{1}{12n^{2}}\right].$$

*Hence*,

$$H(A_{R},\int_{a}^{b}f(t)dt)=H(\left[\frac{1}{2},\frac{4}{3}-\frac{1}{12n^{2}}\right],\left[\frac{1}{2},\frac{4}{3}\right])=\frac{1}{12n^{2}}\leq R\left(n\right)=\frac{1}{4}\sum_{i=1}^{n}h_{i}^{2}{\left\|f{}^{\prime \prime }\right\|}_{\infty }=\frac{1}{2n}.$$

**Fig 5:**
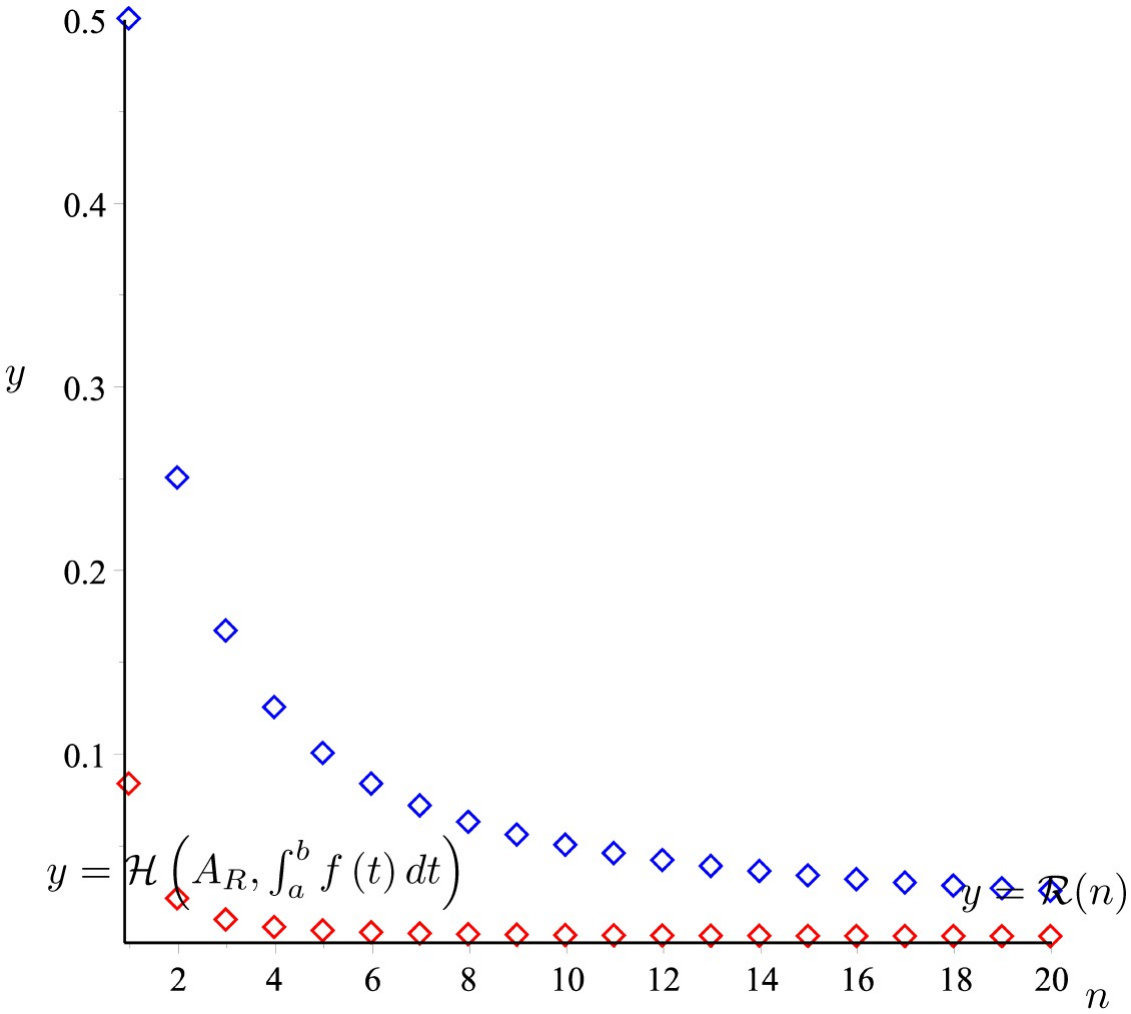
$H(A_{R},\int_{a}^{b}f(t)dt)$ (the red line) and R(n) (the blue line) given in Example 21.

*The graph of the two functions* $H(A_{R},\int_{a}^{b}f(t)dt)$ *and* y=R(n) *is given in* Fig 5. *It is easy to see that*

$$H(A_{R},\int_{a}^{b}f(t)dt)\leq R\left(n\right).$$

*So that the proposition 20 holds*.

**Example 22** *Considering* *f*(*x*) = [*x*^2^, 4*x*^2^], *and* $t_{i}=\frac{i}{n}\left(i=1,2,...,n\right),h_{i}=\frac{1}{n}.$

*We can get* ‖*f*″‖_∞_ = 8, *and*

$$A_{R}=\sum_{i=1}^{n}\;f(\eta_{i})\frac{1}{n},where\;\eta_{i}=\frac{t_{i}+t_{i-1}}{2}.$$

*Thus, we have*

$$A_{R}=\sum_{i=1}^{n}{\left[{\left(\frac{2i-1}{2n}\right)}^{2},4\frac{2i-1}{2n}\right)}^{2}]\cdot \frac{1}{n}=\left[\frac{1}{3}-\frac{1}{12n^{2}},\frac{4}{3}-\frac{1}{3n^{2}}\right].$$

*Hence*,

$$H(A_{R},\int_{a}^{b}f(t)dt)=\frac{1}{3n^{2}}\leq R\left(n\right)=\frac{1}{4}\sum_{i=1}^{n}h_{i}^{2}{\left\|f{}^{\prime \prime }\right\|}_{\infty }=\frac{2}{n}.$$

**Fig 6:**
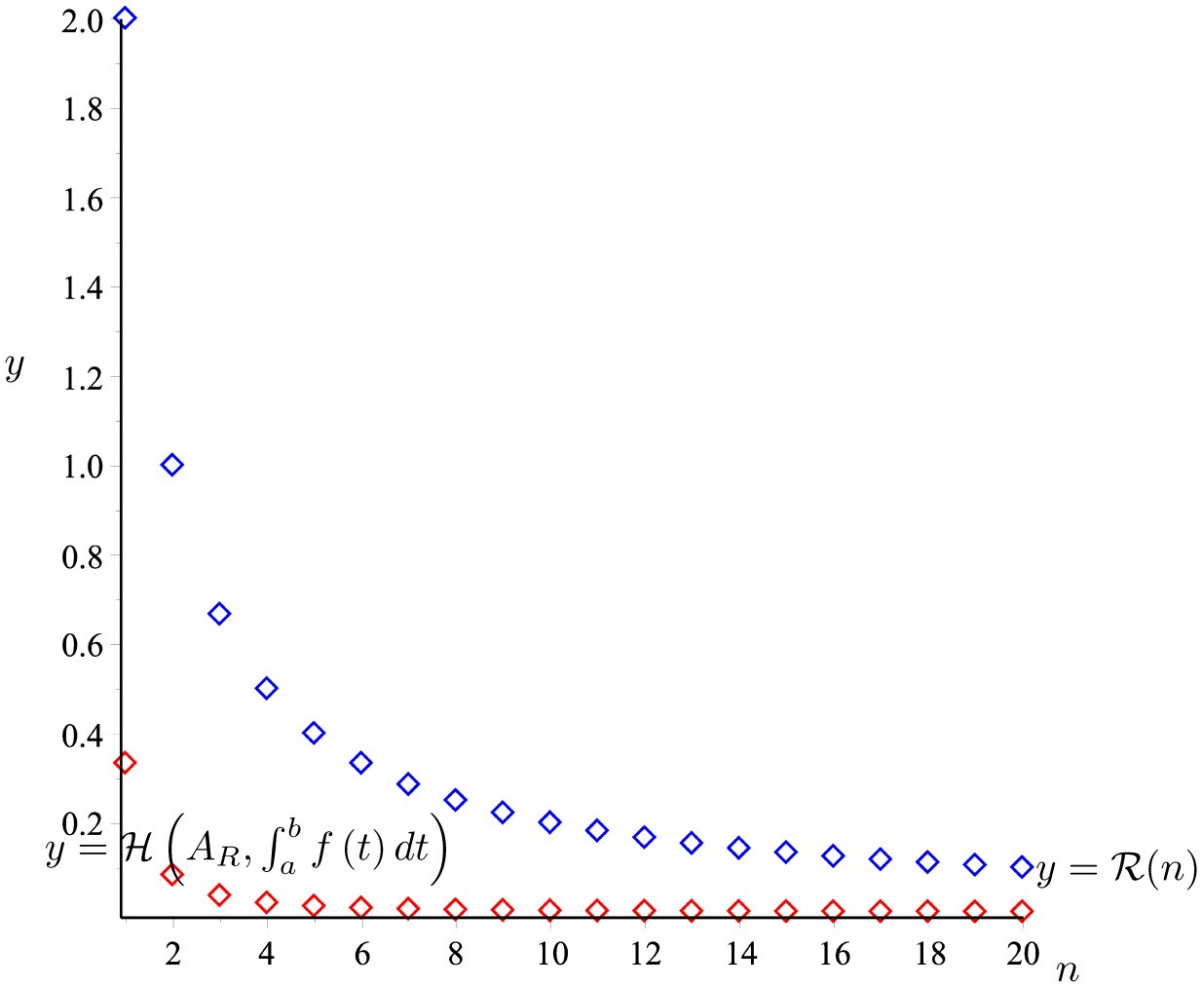
$H(A_{R},\int_{a}^{b}f(t)dt)$ (the red line) and R(n) (the blue line) given in Example 22.

*The graph of the two functions* $H(A_{R},\int_{a}^{b}f(t)dt)$ *and* y=R(n) *is given in* Fig 6. *It is easy to see that*

$$H(A_{R},\int_{a}^{b}f(t)dt)\leq R\left(n\right).$$

*So that the proposition 20 holds*.

**Proposition 23** *Let* $f:\left[a,b\right]\to R_{I}$ *be an interval-value function. If its second order gH-derivative f*″ *is continuous, then for any K-partition* $P\in P(I_{h},\xi )$,

$$H(\int_{a}^{b}f(t)dt,S_{G}(f,P))\leq {\left\|f{}^{\prime \prime }\right\|}_{\infty }\sum_{i=1}^{n}\frac{h_{i}^{2}\left(t_{i}+t_{i-1}\right)}{6},$$

*where S*_*G*_ *denotes the generalized Riemann type quadrature rule defined by*

$$S_{G}(f,P)=\sum_{i=1}^{n}(f(\xi_{i})h_{i}{⊖}_{gH}(\xi_{i}-\frac{t_{i}+t_{i-1}}{2})(f(t_{i}){⊖}_{gH}\;f(t_{i-1}))).$$

*Proof* According to Lemmas 1 and 3 and Corollary 18, we have

$$\begin{array}{c} \;H(\int_{a}^{b}f(t)dt,S_{G}(f,P))\\ =H(\sum_{i=1}^{n}\int_{t_{i-1}}^{t_{i}}f(t)dt,\sum_{i=1}^{n}(f(\xi_{i})h_{i}{⊖}_{gH}(\xi_{i}-\frac{t_{i}+t_{i-1}}{2})(f(t_{i}){⊖}_{gH}\;f(t_{i-1}))))\\ \leq \sum_{i=1}^{n}H(f(\xi_{i})h_{i}{⊖}_{gH}\int_{t_{i-1}}^{t_{i}}f(t)dt,(\xi_{i}-\frac{t_{i}+t_{i-1}}{2})(f(t_{i}){⊖}_{gH}\;f(t_{i-1})))\\ \leq \sum_{i=1}^{n}h_{i}H(f(\xi_{i}){⊖}_{gH}\frac{1}{h_{i}}\int_{t_{i-1}}^{t_{i}}f(t)dt,(\xi_{i}-\frac{t_{i}+t_{i-1}}{2})\frac{f\left(t_{i}\right){⊖}_{gH}\;f(t_{i-1})}{h_{i}})\\ \leq \|f{}^{\prime \prime }{\|}_{\infty }\sum_{i=1}^{n}\frac{h_{i}^{2}\left(t_{i}+t_{i-1}\right)}{6}. \end{array}$$ (25)

The proof is therefore complete.

**Example 24** *Considering* *f*(*x*) = [2*x* + 1, 3*x*^2^ + 4*x* + 2](*x* ∈ [0, 1]), *and* $t_{i}=\frac{i}{n}\left(i=1,2,\ldots ,n\right),h_{i}=\frac{1}{n}$.

*We can get* ‖*f*″‖_∞_ = 6, *and*

$$S_{G}=\sum_{i=1}^{n}\left[\frac{2i-1}{n}+1,\frac{6i^{2}+i+3}{2n^{2}}+\frac{4i-2}{n}+2\right]=\left[2,5+\frac{1}{2n^{2}}\right].$$

*Hence*,

$$\begin{array}{cc} H(\int_{a}^{b}f(t)dt,S_{G}(f,P)) & =H(\left[2,5\right],\left[2,5+\frac{1}{2n^{2}}\right])=\frac{1}{2n^{2}}\\ & \leq R\left(n\right)=\|f{}^{\prime \prime }{\|}_{\infty }\sum_{i=1}^{n}\frac{h_{i}^{2}\left(t_{i}+t_{i-1}\right)}{6}=\frac{1}{n}. \end{array}$$

**Fig 7:**
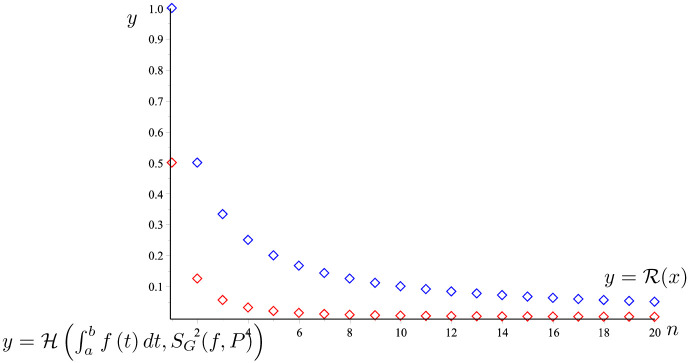
$H(\int_{a}^{b}f(t)dt,S_{G}(f,P))$ (the red line) and R(x) (the blue line) given in Example 24.

*The graph of the two functions* $H(A_{R},\int_{a}^{b}f(t)dt)$ *and* y=R(n) *is given in* Fig 7. *It is easy to see that*

$$H(\int_{a}^{b}f(t)dt,S_{G}(f,P))\leq R\left(n\right).$$

*So that the proposition 23 holds*.

**Example 25** *Considering* *f*(*x*) = [2*x*^2^, 3*x*^3^ + 2*x*^2^ + 6], *and* $t_{i}=\frac{i}{n}\left(i=1,2,\ldots ,n\right),h_{i}=\frac{1}{n}$.

*We can get* ‖*f*″‖_∞_ = 22, *and*

$$\begin{array}{cc} S_{G} & =\sum_{i=1}^{n}\left[2{\left(\frac{i}{n}\right)}^{2},3{\left(\frac{i}{n}\right)}^{3}+2{\left(\frac{i}{n}\right)}^{2}+6\right]\frac{1}{n}\\ & \;\;\;\;\;\;\;\;\;\;\;\;{⊖}_{gH}{\left[2{\left(\frac{i}{n}\right)}^{2}-2{\left(\frac{i-1}{n}\right)}^{2},3{\left(\frac{i}{n}\right)}^{3}-\frac{i-1}{n}\right)}^{3})+2{\left(\frac{i}{n}\right)}^{2}-\frac{i-1}{n}{)}^{2})]\\ & =\left[2,5+\frac{1}{2n^{2}}\right]\frac{1}{2n}. \end{array}$$

*Hence*,

$$\begin{array}{cc} H(\int_{a}^{b}f(t)dt,S_{G}(f,P)) & =H(\left[\frac{2}{3},\frac{89}{12}\right],\left[\frac{2}{3}+\frac{1}{3n^{2}},\frac{89}{12}+\frac{13}{12n^{2}}\right])=\frac{13}{12n^{2}}\\ & \leq R\left(n\right)=\|f{}^{\prime \prime }{\|}_{\infty }\sum_{i=1}^{n}\frac{2i-1}{6n^{3}}=\frac{11}{3n}. \end{array}$$

**Fig 8:**
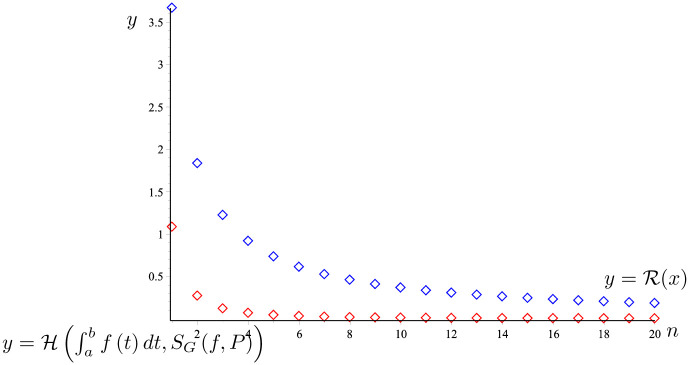
$H(\int_{a}^{b}f(t)dt,S_{G}(f,P))$ (the red line) and R(x) (the blue line) given in Example 25.

*The graph of the two functions* $H(A_{R},\int_{a}^{b}f(t)dt)$ *and* y=R(n) *is given in* Fig 8. *It is easy to see that*

$$H(\int_{a}^{b}f(t)dt,S_{G}(f,P))\leq R\left(n\right).$$

*So that the proposition 23 holds*.

## Conclusion

This article presents a novel application of Ostrowski and Čebyšev type inequalities to gH-differentiable interval-valued functions, expanding upon the existing theory. The findings of this study contribute significantly to the field of interval differential (or integral) inequalities and interval differential equations. By incorporating generalized Hukuhara differentiability and introducing interval-based variations of Ostrowski and Čebyšev type inequalities, our work advances the field of interval analysis, providing researchers and practitioners with powerful tools to address the complexities associated with interval-valued functions. Several related examples are solved to demonstrate the proposed method’s effectiveness, showcasing its practical applicability. Additionally, this article introduces new error estimation techniques for fuzzy quadrature rules, showcasing a theoretical application of these mathematical techniques. In scenarios where the functions $\underline{f}$ and $\bar{f}$ lack differentiability, conventional error estimation methods for quadrature rules must be revised. Classical error estimation results, which rely on differentiability assumptions, are not applicable in these cases. However, by considering interval-valued functions and extending existing quadrature formulas for real functions, the approximation of $\int_{a}^{b}f\left(t\right)dt$ becomes feasible. Consequently, these extended quadrature formulas provide a valuable means of approximating the integral when dealing with interval-valued functions.

Furthermore, there are additional opportunities for further development and exploration. For instance, new versions of Ostrowski and Čebyšev type inequalities can be derived for various unique means, such as arithmetic mean, geometric mean, harmonic mean, and so on [17]. In future research, it is intended to utilize these new and exciting inequalities for fuzzy-interval-valued functions. Additionally, it is anticipated that applying Ostrowski and Čebyšev type inequalities to set-valued and fuzzy set-valued functions will have implications in the fields of fluctuations, dynamic systems, hesitant fuzzy sets, and other related areas.

## References

[1] Čebyšev P. L., Sue les expressions approximatives des intégrales définies par les autres prises entre les mêmes limites, Proc. Math. Soc. Charkov, 2 (1882) 93–98.

[2] Ostrowski A. M., Über die absolutabweichung einer differentiebaren funktion van ihrem integralmittewert, Comment Math. Helv., 10 (1) (1938) 226–227. doi: 10.1007/BF01214290

[3] D. S. Mitrinović, Analytic Inequalities. (In cooperation with P.M. Vasić) Die Grundlehren der mathematischen Wissenschaften, Band 165, Springer-Verlag, New York-Berlin, 1970.

[4] Mitrinović D. S., Vasić P. M., History, variations and generalisations of theČebyšev inequality and the question of some priorities, Univ. Beograd. Publ. Elektrotehn. Fak. Ser. Mat. Fiz. No. 461–497 (1974), 1–30.

[5] Milovanović G. V., Pečarić J. E., On generalization of the inequality of A. Ostrowski and some related applications, Univ. Beograd. Publ. Elektrotehn. Fak. Ser. Mat. Fiz. No. 544–576 (1976), 155–158.

[6] Milovanović G. V., Ostrowski type inequalities and some selected quadrature formulae, Appl. Anal. Discrete Math. 15 (2021), no. 1, 151–178. doi: 10.2298/AADM200609054M

[7] Irshad N., Khan A.R., Mehmood F., Pečarić J., New Perspectives on the Theory of Inequalities for Integral and Sum, Birkhäuser Cham, 2022.

[8] Pachpatte B. G., Analytic inequalities: recent advances, Atlantis Press, 2012.

[9] Grüss G., Über das maximum des absoluten betragts von $\frac{1}{b-a}\int_{a}^{b}f\left(x\right)g\left(x\right)dx-\frac{1}{{\left(b-a\right)}^{2}}\int_{a}^{b}f\left(x\right)dx\int_{a}^{b}g\left(x\right)dx$, Math. Z., 39 (1) (1935), 215–226.

[10] Ujević N., Sharp inequalities of Simpson type and Ostrowski type, Computers and Math. Appl., 48 (2004) 145–151.

[11] Acu A. M., Improvement of Grüss and Ostrowski type inequalities, Filomat, 29 (9) (2015) 2027–2035. doi: 10.2298/FIL1509027A

[12] Agahi H., Mohammadpour A., Mesiar R., Generalizations of the Chebyshev-type inequality for Choquet-like expectation, Inf. Sci., 236 (2013) 168–173. doi: 10.1016/j.ins.2013.02.019

[13] Cerone P., Dragomir S. S., Some new Ostrowski-type bounds for the Čebyšev functional and applications, J. Math. Inequal., 8 (1) (2014) 159–170. doi: 10.7153/jmi-08-10

[14] Costa T. M., Flores-Franulic A., Chalco-Cano Y., Aguirre-Cipe I., Ostrowski–type inequalities for fuzzy-valued functions and its applications in quadrature theory, Inf. Sci., 529 (2020) 101–115. doi: 10.1016/j.ins.2020.04.037

[15] Dragomir S. S., weighted integral inequalities of Ostrowski, Čebyšev and lupas type with applications, Bull. Aust. Math. Soc., 98 (3) (2018) 439–447. doi: 10.1017/S0004972718000801

[16] Hussain S., Generalization of Ostrowski and Čebyšev type inequalities involving many functions, Aequat. Math., 85 (2013) 409–419. doi: 10.1007/s00010-012-0142-1

[17] Kiris M. E., Sarikaya M. Z., On Ostrowski type inequalities and Čebyšev type inequalities with applications, Filomat, 29 (8) (2015) 1695–1713. doi: 10.2298/FIL1508695K

[18] Liu W., New bounds for the companion of Ostrowski’s inequality and applications, Filomat, 28 (1) (2014), 167–178. doi: 10.2298/FIL1401167L

[19] Masjed-Jamei M., Dragomir S. S., A generalization of the Ostrowski-Gruss inequality, Anal. Appl., 12 (2) (2014) 117–130. doi: 10.1142/S0219530513500309

[20] Qayyum A., Faye I., Shoaib M., Improvement of Ostrowski integral type inequalities with application, Filomat, 30 (6) (2016) 1441–1456. doi: 10.2298/FIL1606441Q

[21] Abbaszadeh S., Gordji M. E., Pap E., Szakái A., Jensen-type inequalities for Sugeno integral, Inf. Sci. 376 (2017) 148–157. doi: 10.1016/j.ins.2016.10.006

[22] Agahi H., Mesiar R., Ouyang Y., General Minkowski type inequalities for Sugeno integrals, Fuzzy Sets Syst. 161 (2010) 708–715. doi: 10.1016/j.fss.2009.10.007

[23] Pap E., Štrboja M., Generalization of the Jensen inequality for pseudo-integral, Inf. Sci. 180 (2010) 543–548. doi: 10.1016/j.ins.2009.10.014

[24] Wang R. S., Some inequalities and convergence theorems for Choquet integral, J. Appl. Math. Comput. 35 (1) (2011) 305–321. doi: 10.1007/s12190-009-0358-y

[25] Aumann R. J., Integrals of set-valued functions, J. Math. Anal. Appl. 12 (1965) 1–12. doi: 10.1016/0022-247X(65)90049-1

[26] Klein E., Thompson A. C., Theory of Correspondences, A Wiley-Interscience Publication, New York, 1984.

[27] Costa T. M., Román-Flores H., Some integral inequalities for fuzzy-interval-valued functions, Inf. Sci., 420 (2017) 110–125. doi: 10.1016/j.ins.2017.08.055

[28] Khan M. B., Zaini H. G., Treanǎ S., Soliman M.S., Nonlaopon K., Riemann–liouville fractional integral inequalities for generalized pre-invex functions of interval-valued settings based upon pseudo order relation, Mathematics, 10(2) (2022) 1–17. doi: 10.3390/math10020204

[29] Budak H., Tun T., Sarikaya M. Z., Fractional hermite-hadamard-type inequalities for interval-valued functions, Proceedings of the American Mathematical Society,148(2) (2019) 705–718. doi: 10.1090/proc/14741

[30] Zhao D., An T., Ye G., Liu W., Chebyshev type inequalities for interval-valued functions, Fuzzy Sets Syst., 396 (2020) 82–101. doi: 10.1016/j.fss.2019.10.006

[31] Zhao D., An T., Ye G., Liu W., Some generalizations of opial type inequalities for interval-valued functions, Fuzzy Sets Syst., 436 (2021) 128–151. doi: 10.1016/j.fss.2021.03.017

[32] Zhao D., Zhao G., Ye G., Liu W., & Dragomir S. S., On hermite–hadamard-type inequalities for coordinated h -convex interval-valued functions, Mathematics, 9(19) (2021), 1–14. doi: 10.3390/math9192352

[33] Budak H., Kashuri A., Butt S., Fractional Ostrowski type inequalities for interval valued functions, Mathematics, 36(8) (2022), 2531–2540.

[34] Khan M. B., Abdullah L., Noor M. A. et al, New hermite–hadamard and jensen inequalities for log-h-convex fuzzy interval valued functions, International Journal of Computational Intelligence Systems, 14 (2021), 155. doi: 10.1007/s44196-021-00004-1

[35] Anastassiou G. A., Fuzzy ostrowski type inequalities, Computational & Applied Mathematics, 22(2) (2003), 279–292.

[36] Bede B., Gal S. G., Generalizations of the differentiability of fuzzy-number-valued functions with applications to fuzzy differential equations, Fuzzy Sets and Systems, 151(3) (2005), 581–599. doi: 10.1016/j.fss.2004.08.001

[37] Chalco-Cano Y., Román-Flores H., Jiménez-Gamero M. D., Generalized derivative and *π*-derivative for set-valued functions, Information Sciences, 181(11) (2011), 2177–2188. doi: 10.1016/j.ins.2011.01.023

[38] Chalco-Cano Y., Román-Flores H., Jiménez-Gamero M. D., Ostrowski type inequalities for interval-valued functions using generalized Hukuhara derivative, Computational & Applied Mathematics, 181(11) (2011), 2177–2188.

[39] Chalco-Cano Y., Rufian-Lizanab A., Román-Flores H., Jiménez-Gamero M. D., Calculus for interval-valued functions using generalized hukuhara derivative and applications, Fuzzy Sets & Systems, 219(16) (2013), 49–67. doi: 10.1016/j.fss.2012.12.004

[40] Stefanini L., Bede B., Generalized Hukuhara differentiability of interval-valued functions and interval differential equations, Nonlinear Anal, 71 (2009) 1311–1328. doi: 10.1016/j.na.2008.12.005

[41] Stefanini L., A generalization of Hukuhara difference and division for interval and fuzzy arithmetic, Fuzzy Sets Syst., 161 (2010) 1564–1584. doi: 10.1016/j.fss.2009.06.009

[42] Chalco-Cano Y., Costa T. M., Román-Flores H., Rufián-Lizana A., New properties of the switching points for the generalized Hukuhara differentiability and some results on calculus, Fuzzy Sets Syst., 404 (2021), 62–74. doi: 10.1016/j.fss.2020.06.016

[43] Milovanović G.V., On some integral inequalities, Univ. Beograd. P Elektrotehn. Fak. Ser. Mat. Fiz. No. 498–541 (1975), 119–124

[44] Franjić I., Pčečari J., Perić I., Vukelić A., Ana Euler integral identity, quadrature formulae and error estimations (from the point of view of inequality theory). ELEMENT, Zagreb, 2011.

[45] R. E. Moore, M. J.Cloud, Computational functional analysis, Halsted Press, 1985.

[46] Moore R. E., Kearfott R. B., Cloud M. J., Introduction to Interval Analysis, Society for Industrial and Applied Mathematics, 2009.

[47] Goestschel R., Voxman W., Elementary fuzzy calculus, Fuzzy Sets Syst., 18 (1986) 31–43. doi: 10.1016/0165-0114(86)90026-6

[48] Chalco-Cano Y., Flores-Franulic A., Román-Flores H., Ostrowski type inequalities for interval-valued functions using generalized Hukuhara derivative, Comput. Appl. Math., 31 (3) (2012) 457–472.

[49] Chalco-Cano Y., Lodwick W. A., Condori-Equice W., Ostrowski type inequalities and applications in numerical integration for interval-valued functions, Soft Comput., 19(11) (2015) 3293–3300. doi: 10.1007/s00500-014-1483-6

